# Analysis of Human RSV Immunity at the Molecular Level: Learning from the Past and Present

**DOI:** 10.1371/journal.pone.0127108

**Published:** 2015-05-22

**Authors:** Kerrie Vaughan, Julia Ponomarenko, Bjoern Peters, Alessandro Sette

**Affiliations:** 1 La Jolla Institute for Allergy and Immunology, La Jolla, California, United States of America; 2 San Diego Supercomputer Center, University of California San Diego, La Jolla, California, United States of America; University of Iowa, UNITED STATES

## Abstract

Human RSV is one of the most prevalent viral pathogens of early childhood for which no vaccine is available. Herein we provide an analysis of RSV epitope data to examine its application to vaccine design and development. Our objective was to provide an overview of antigenic coverage, identify critical antibody and T cell determinants, and then analyze the cumulative RSV epitope data from the standpoint of functional responses using a combinational approach to characterize antigenic structure and epitope location. A review of the cumulative data revealed, not surprisingly, that the vast majority of epitopes have been defined for the two major surface antigens, F and G. Antibody and T cell determinants have been reported from multiple hosts, including those from human subjects following natural infection, however human data represent a minority of the data. A structural analysis of the major surface antigen, F, showed that the majority of epitopes defined for functional antibodies (neutralizing and/or protective) were either shown to bind pre-F or to be accessible in both pre- and post-F forms. This finding may have has implications for on-going vaccine design and development. These interpretations are in agreement with previous work and can be applied in the larger context of functional epitopes on the F protein. It is our hope that this work will provide the basis for further RSV-specific epitope discovery and investigation into the nature of antigen conformation in immunogenicity.

## Introduction

Despite the fact that viral pathogenesis and immunity to RSV are well characterized in humans and in animal models of disease [1–4], human respiratory syncytial virus (HRSV) is one of the most prevalent viral pathogens of early childhood for which no vaccine is available. In the 1960s, a formalin-inactivated (FI-RSV) vaccine candidate failed to provide protection and was associated with enhanced disease [5]. As a result, subsequent research focused on elucidating the underlying mechanisms of disease enhancement and clarifying correlates of protection to ensure that future vaccine candidates would be uniformly effective.

It is believed that FI-RSV vaccine enhanced disease was a result of the alteration of critical epitopes as a result of formalin inactivation [6–9]. The generation of non-neutralizing and non-fusion inhibiting antibodies and the predominance of an inflammatory CD4^+^ T cell-driven Th_2_ cytokine response [10–12] have been shown to play roles in disease exacerbation. Indeed, chemical alteration of epitopes has been implicated in immune responses to several vaccine candidates, including measles [13, 14], influenza A [15] and pertussis [16, 17]. Understanding the molecular mechanisms of protection and disease exacerbation in the context of both humoral and cellular responses helps guide current efforts towards vaccine development.

Several groups are pursuing novel approaches for RSV vaccine design that incorporate epitope- and structure-based methods [18–21]. These approaches focus on the fusion protein (F), a major target of neutralizing antibodies [8, 22]. The F protein is a prominent surface glycoprotein that mediates viral attachment, penetration and viral spread. During infection, F undergoes significant processing, structural and conformational changes. The translated F_0_ precursor is cleaved into two disulfide-linked chains, F_2_ and F_1_ [23, 24]. The F protein exists *in vivo* in two configurations: a globular pre-fusion (pre-F) form present in virus particles [25], which is triggered upon binding to the host cell to refold into an elongated post-fusion (post-F) form. It is believed that the pre-fusion form is a major target of neutralizing antibody activity [26]. However well-known monoclonal antibodies used prophylactically in humans [e.g. Palivizumab (Synagis)] have been shown to bind the post-fusion F protein [18, 27]. This is explained by the fact that some antibody epitopes remain accessible and unchanged in both pre- and post-F fusion forms [19, 28].

Our aim was to use the data and tools housed in the Immune Epitope Database (IEDB) and Analysis resource [www.epitope.org] to comprehensively analyze all antibody/B cell and T cell epitopes described to date for the F protein. We used a combination of *in silico* prediction tools housed at the IEDB and other computational methods to characterize the nature and structural features of these epitopes on available 3D structures of the F protein and then compared these results to known (experimentally derived) functional/protective sites. We found that the majority of epitopes defined for functional antibodies was either shown to bind pre-F or to be solvent accessible in both pre- and post-F forms. Finally, we observed an alignment of functional B and T cell epitopes mapped along the length of the F protein, which might suggest a relationship between the location of epitopes and protein functional sites. These findings are in agreement with previous works [18–20] and can be applied in the larger context of all reported functional epitopes on the RSV F protein.

## Materials and Methods

### Data Queries to the IEDB

All queries were performed using the Immune Epitope Database and Analysis Resource (IEDB) home page search interface [www.iedb.org]. For more complex queries the advanced search interface (all fields) was utilized. Results were downloaded in Excel format for detailed analysis. Excel spreadsheets containing all B and T cell assay data were used to identify epitope sequence and source, assay type, immunization details, antibody isotype, effector cells (CD4/CD8), host, etc. RSV-specific queries were conducted using the Antigen organism finder, using the input ‘Pneumovirus.’ In the IEDB, this genus currently includes human RSV (HRSV, RSV), bovine RSV (BRSV) and Ovine RSV (ORSV). The latter strains are used as animal models of natural infection and the viruses are antigenically homologous. Antigen-specific queries were performed using Antigen Finder or through analysis of above spreadsheets for response-association. Unless otherwise indicated, all reported data herein represent positive epitopes and/or assays only.

### Nature and scope of assays defining epitopes

The IEDB defines an epitope the unique molecular structures (minimal sequences, linear and discontinuous regions, as well as key residues) experimentally shown to react with a B cell or T cell (no predictions). This includes peptides less than or equal to 50 amino acids in size and non-peptidic structures less than or equal to 5000 Daltons. These structures must be experimentally tested for binding to an adaptive immune receptor (T cell receptor (TCR), antibody or B cell receptor (BCR), or major histocompatibility complex (MHC)) or the receptor must be known and stated to be epitope specific in order to be included in the IEDB. Positive epitopes are defined as those residues or structures shown to bind in at least one positive assay; negative data (residues shown to be non-binding) are also captured in the IEDB. With respect to assay type, B cell/antibody assays considered herein to define ‘functional epitopes’ (Table 1) include, *in vitro* neutralization (microneutralization or plaque reduction), and *in vivo* protection assays whereby epitope immunization is shown to increase survival or protection (decreased symptoms or viral load) following live viral challenge in an animal model. T cell assays considered to define functional epitopes include *in vivo* protection as described above, and those assays demonstrating *in vitro* cytotoxicity [^51^chromium release or non-radioactive assay] showing epitope-specific killing of target cells, as well as *in vitro* cytokine production [ELISPOT, ELISA, and flow cytometry], wherein peripheral blood mononuclear cells (PBMCs) or lymphocytes are stimulated with or respond to the epitope as assay antigen.

**Table 1:** Linear B cell epitopes associated with virus neutralization or *in vivo* protection.

Epitope	Residues	Source	Antibody	Assay	Assay Ag	PMID
SKPTTKQRQNKPPNKP	144–159	G HRSV Long, A2	PC	Prot, VN	Long^X^	11257359, 1720589
QNKPPNKPNNDF	152–163	G HRSV Long	5C2 (IgG1)	Prot _(PT)_	Long	11257359
HFEVFNFVPCSIC	164–176	G HRSV Long	PC	Prot	Long	11257359
FEVFNFVP	165–172	G HRSV Long	5B7 (IgG1)	Prot_(PT)_	Long	11257359
FVPCSICSNNPTCWAICKRIP	170–190	G HRSV A2	PC	Prot	A2	11053254
VPCSICSNNPTCWAICK	171–187	G HRSV Long	PC;18D1	Prot_(PT)_	Long, RSV B 8/60^X^	9141214, 11257359
PCSICSNNPTCWAICK	172–187	G HRSV	PC	Prot	HRSV	11053254
CSICSNNPTCWAICK	173–187	G HRSV Long	PC	Prot	Long	9234952
STCEGNLACLSL	174–185	G BRSV Snook	PC	Prot	BRSV (NI)	23890818
KRIPNKKPGKKTTT	187–200	G HRSV Long	PC	VN	Long	1383397
PNKKPGKKTTTKPTK	190–204	G HRSV Long	MS	Prot_(PT)_	Long	11257359
KKTTTKPTK	196–204	G HRSV Long	8A3	Prot(_PT)_	Long	11257359
IPELIHYTRNSTKRFYGLMGKKRKRRFLGFLLGIGSAI	111–148	F BRSV (RB94)	PC	Prot	BRSV (NI)	23890818
STNKAVVSLS	173–182	F BRSV (RB94)	3	VN	BRSV Lelystad,^X^ Long	9541615
PIVNKQSCSISNIETVIEFQQ	205–225	F HRSV Long, A2; F HRSV B (18537)	PC	VN	Long, HRSV B^X^ (18537); HRSV 9320	2033389, 7685537, 9228999
IEFQQKNNRLLEITREF	221–237	F HRSV A2	PC	Prot	A2	1706591
STYMLTNSELLSLINDMPITNDQKKLMSNNVQIVRQ	248–283	F HRSV	**Motavizumab** (IgG1)	VN	A2	20943340
NSELLSLINDMPITNDQKKLMSNN	254–277	F HRSV A2	**Motavizumab** (IgG1)	VN	A2	20098425, 23618766
SELLSLINDMPITNDQKKLMSNNV	255–278	F HRSV Long	PC	VN, Prot	Long	17603843, 7685537
TASNKNRGIIKTFS	423–436	F HRSV A2	ch101F^	VN	A2	17872524
KNRGIIKTFSN	427–437	F HRSV A2	**101F** (IgG2a)	VN	A2	20881049, 23618766

PT, passive transfer

^X^, shows cross-protection; PC, polyclonal; VN, virus neutralization; Prot, *in vivo* challenge/survival; NI, natural infection

^ch101F is a mouse-human chimeric of 101F; **Motavizumab** is also a mouse-human chimera; all others are mouse; A2, HRSV A2 strain; Long, HRSV long strain

Also of note with respect to the source of the data presented herein, discontinuous epitopes (non-linear/conformational) were most frequently defined using virus escape mutants whereby the alteration or substitution of critical residues conferred escape from *in vitro* neutralization in a standard microneutralization assay. Epitopes captured to date were also defined by crystallographic analysis of antigen-antibody complexes (x-ray crystallography) [PDBs: 3IXT, 3QWO, 4JLR (motavizumab), 4JHW (D25), 4N9G (17HD9), 3O41, 3O45 (101F)].

### Pre- and Post-fusion Structures Analysis

For direct comparisons of antibody epitope prediction scores, Protein Data Bank (PDB) [www.rcsb.org/pdb] structures for the pre-F [PDB: 4HJW, 4MMS] and post-F [PDB: 3RKI, 3RRR] conformations were used. Separate chains comprising the trimeric structure of the F protein were re-worked manually into a single chain, which was used as input for the B cell epitope prediction tools, ElliPro [29] and Discotope (available on IEDB) [30, 31], both housed in the IEDB. Residue solvent accessibility in the structures was calculated with NACCESS [32]. We considered relative solvent accessibility (RSA) for side-chain atoms. Residue evolutionary conservation was calculated with the ConSurf server [33], using the multiple sequence alignment (MSA) generously provided by Dr. Jason S. McLellan [19].

For each predicted epitope in each protein, we calculated the correctly (true positive) and incorrectly predicted epitope residues (false positive) and non-epitope residues, which were defined as all other protein residues (true negative and false positive). The statistical significance of a prediction, that is, the difference between observed and expected frequencies of an actual epitope/non-epitope residue in the predicted epitope/non-epitope, was determined using the Fisher's exact test. The prediction was considered significant if p-value was < = 0.05.

### Immunome Browser and Homology Mapping Tool

The Immunome Browser (IB) is tool available on the IEDB website that allows users to visualize the relative prominence of antibody and T cell epitopes on their derivative antigens. The IB plots the response frequency score for each residues of an existing epitope onto an antigen or reference proteome. The response frequency score (RFscore) is calculated as (responded-square root (responded))/tested, where “tested” and “responded” correspond to numbers of individuals tested and responded to a given residue. The score has a range [0 to 1], and a higher score indicates that a larger fraction of individuals responded. The square root is a correction factor, approximating one standard deviation for the number of responding donors. This gives a higher score to epitopes studied with larger sample sizes. [34]. Thus, the tool allows visualizing those regions on the antigen(s) that are more immunodominant or more frequently studied in a given population for a particular response (Ab, T, CD4, CD8, etc.). This provides the least biased way to analyze the cumulative data and compare immune response among hosts, disease states, and assay types; for example, neutralization assays versus protection assays.

### Homology Mapping

The Homology Mapping tool can be accessed at http://tools.immuneepitope.org/esm/userMappingFrontP.jsp or through the IEDB Analysis Tools menu. This tool uses input from the user (epitope residues or epitope ID) to map individual or a group of epitopes onto the three dimensional structure of the source protein provided by entering a SWISS-PROT, GenBank ID or FASTA. JAVA version 7 is required for this application.

## Results

### Summary of RSV immune epitope data

To determine the breadth of RSV-specific data available in the IEDB, we performed a broad query to enumerate all data reported from viruses within the genus *Pneumovirus*. As of January 2015, the IEDB contained 140 references describing 672 epitopes derived from this genus, including human RSV (567), bovine RSV (86), murine pneumonia virus (19) and ovine RSV (1) [number in parentheses means total epitopes for each virus]. These data include antibody/B cell (243) and T cell (323) epitopes, as well as epitopes naturally-eluted from human MHC-peptide complexes and those tested in MHC binding assays (160). Of the B cell epitopes, linear epitopes (208) vastly outnumbered discontinuous (35). For T cell epitopes, the majority have been described for class II/CD4^+^ (206) compared to class I/CD8^+^ (97). Thus the majority of reported data were from human RSV, representing a fairly broad range of immune reactivities, including all those within the broad categories of assay types: binding assays (e.g., ELISA), functional assays (e.g., neutralization, CTL) and *in vivo* challenge assays. Of the total T and B cell epitopes reported to date, 214 epitopes (32%) have been defined in humans. Non-human animal models used in the assays identifying RSV epitopes included mice (307), cows (88), rabbits (39), guinea pigs (2), rhesus monkeys (2) and Cynomolgus monkeys (1).

**Fig 1:**
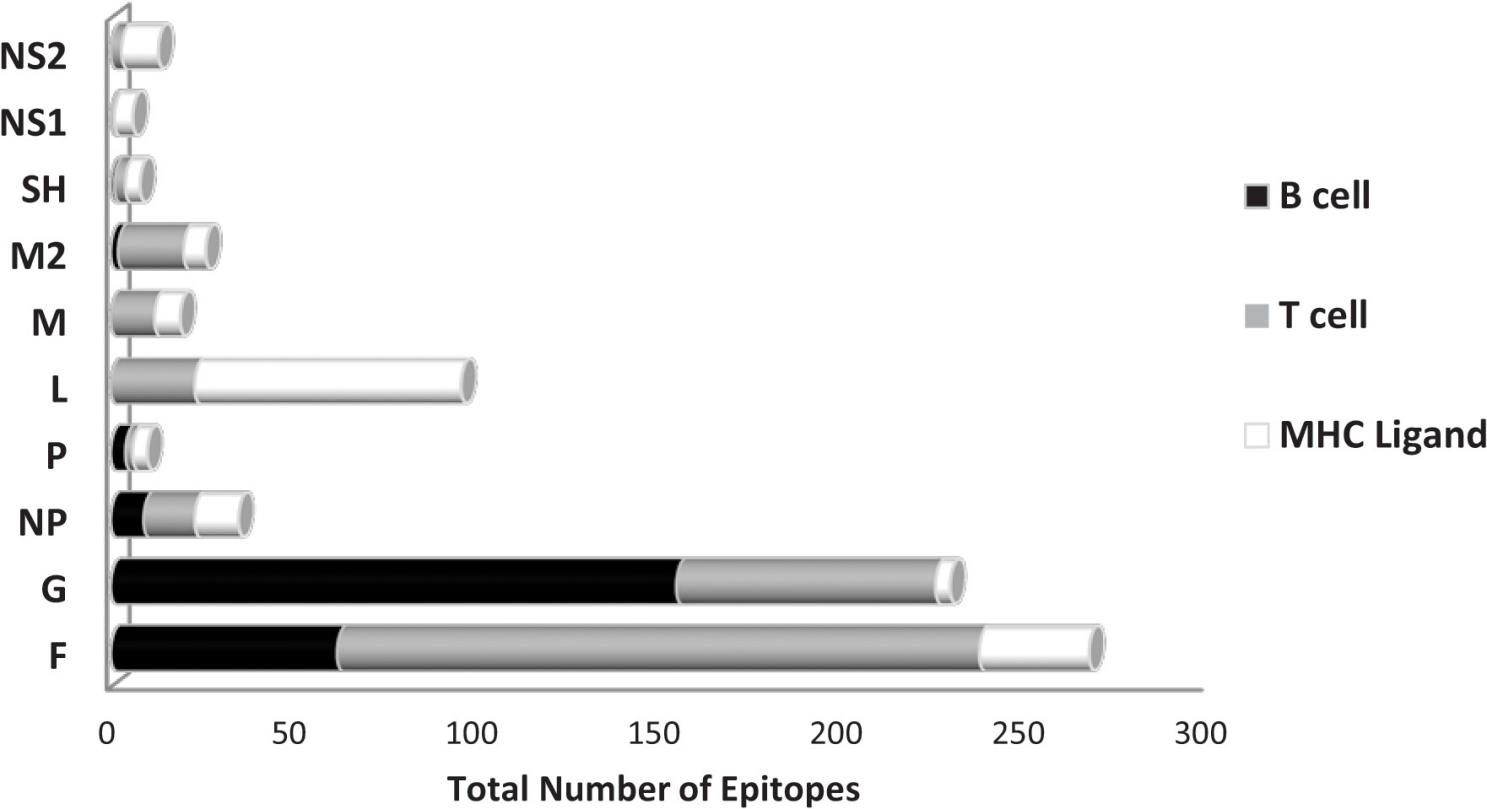
Response by RSV protein. The total number of unique epitopes reported to date for each RSV protein are shown. Data are then further broken down into response type, antibody/B cell (black), T cell (grey) and MHC ligand (elution and binding combined; white). The percentages provided represent the portion of the total RSV-specific data.

Epitopes have been reported for all ten RSV protein antigens: fusion (F) glycoprotein, attachment (G) glycoprotein, RNA-dependent RNA polymerase (L), nucleoprotein (NP), matrix (M), matrix 2 (M2), non-structural protein 1 (NS1), non-structural protein 2 (NS2), phosphoprotein (P) and the small hydrophobic protein (SH). However, the degree of coverage varied greatly among individual antigens. Not surprisingly, the major surface glycoproteins, F and G, represented the majority of identified epitopes (37% and 35%, respectively), followed by L (12%), NP (5%), M2 (4%), M (3%), NS2 (2%), P (1%), SH (1%) and NS1 (1%). Fig 1 shows the distribution of epitopes for each RSV antigen and further illustrates the relative proportion of response types (antibody/B cell, T cell or MHC ligand binding/elution) for each antigen. At the level of antibody responses, epitopes from F and G clearly predominated. The other antigens had far fewer total reported epitopes, and they were primarily associated with T cell responses and/or MHC binding.

### Functional analysis of the humoral response to the predominant surface antigens F and G

The predominance of epitopes derived from F and G was not unexpected, as these antigens are well-documented as the major targets of immune response to RSV infection [22]. In particular, F has received significant attention in the characterization of RSV immunity [e.g. Motavizumab (Numax), palivizumab (Synagis), RSV SAM (self-amplifying message) vaccine, RSV-IGIV (RespiGam), [35] due to its ability to induce neutralizing antibodies and to play a putative role in protection from RSV.

To characterize in depth the B cell/antibody data for the F and G proteins, we focused on the subset of data describing functional assays, including only data related to *in vivo* protection assays and those defining *in vitro* correlates of protection, virus neutralization/fusion-inhibition (described in the Methods). From here forward, we will refer to this specific subset of data as ‘functional epitopes.’ Many epitope mapping studies are carried out by testing overlapping 15-20-mer peptides spanning the entire length of an antigen. While this is a standard approach for epitope mapping, the interaction it defines is less frequently indicative of a biologically relevant activity. Assays that defined binding alone have therefore have been excluded.

Tables 1 and 2 provide a summary of all linear and discontinuous B cell epitopes associated with virus neutralization and/or *in vivo* protection, respectively. Data for linear epitopes include epitopes identified in assays using both polyclonal sera (PC) and monoclonal antibodies (mAbs). Discontinuous epitopes were defined for both human and murine mAbs, and include well-known mAb D25 [35], palivizumab (humanized murine mAb derived from mAb 1129), and motavizumab (a second-generation humanized mAb derived from palivizumab) [36–38]. Some of the epitopes were identified via protection following passive transfer of epitope-specific antibodies or survival frequency (number protected/number tested), and some were associated with cross-protection (response to other strains or to BRSV). It is important to note that some epitopes, which were originally described as linear, were subsequently further refined for specificity as discontinuous epitopes (e.g., motavizumab epitope). It is also of note that only three of these mAbs were defined in human hosts (D25, Fab19 and MPE8), while the remainder were derived from murine hosts.

**Table 2:** Discontinuous B cell epitopes associated with virus neutralization or *in vivo* protection.

Epitope	Source	Antibody	Assay	Assay Antigen	PMID
Q142, K145	G HRSV A Mon/3/88	021/5G (IgG1)	VN	HRSV A Mon/3/88	9349460
F163, F165	G HRSV A Mon/3/88	021/1G (IgG2a)	VN	HRSV A Mon/3/88	9349460
T181	G HRSV A Mon/3/88	021/19G (IgG1)	VN	HRSV A Mon/3/88	9349460
R188	G HRSV A Mon/3/88	021/18G,021/2G, 021/17G, 021/20G, 021/22G	VN	HRSV A Mon/3/88	9349460
R188, K192	G HRSV Long	PC	Prot, VN	HRSV Long	12744884
R244	G HRSV A Mon/3/88	021/9G, 021/4G, 021/16G (IgM)	VN	HRSV A Mon/3/88	9349460
T282, L286	G HRSV A Mon/3/88	021/7G (IgG1)	VN	HRSV A Mon/3/88	9349460
F32, K272	F HRSV A2	1142	VN	HRSV A2	9878616
T50, L305, G307, I309, D310	F HRSV A2	MPE8*	VN, Prot	HRSV A2, Long, HRSV B, HMV, PVM, BRSV (RB94)^*^	23955151
N63, K65, E66, K68, K196, N197, Y198, I199, D200, K201, Q202, L203, L204, P205, I206, V207, N208, K209, Q210	F HRSV A2	D25 (IgG1)*	VN	HRSV A2	23618766
S190, L258, K272	F HRSV Long	**7C2**	VN	HRSV Long, A2^*^	1383404
N216, N262, N268, K272	F HRSV Long	**47F, AK13A2**	VN	HRSV Long, A2*	1383404
A241, K421	F HRSV A2	1308F, 1302A	VN	HRSV A2	9878616
L258, S259, N262, D263, M264, P265, I266, T267, N268, D269, K271, K272, S275	F HRSV	17HD9**	VN	HRSV B	24499818
N262	F HRSV A2	**1153**	VN	HRSV A2	9878616
N262, N268	F HRSV Long	**47F**	VN	HRSV Long	1688629
I266	F HRSV A2	**Fab 19** (IgG1)*	VN	HRSV A2	9878616
N268	F HRSV Long	**11**	VN	HRSV Long, A2^*^	1383404
S275	F HRSV A2	**1129**	VN	HRSV A2	9878616
K272, S275	F HRSV A2	**Palivizumab**	VN	HRSV	17623075
S255, L258, S259, N262, N268, D269, K271, K272, S275, N276	F HRSV	**Motavizumab**	VN	HRSV	21549714
K272	F HRSV A2	**151, 1200**	VN	HRSV A2	9878616
N276	F HRSV A2	**1237**, **1214**	VN	HRSV A2	9878616
P389	F HRSV A2	1269, 131-2a, 55F	VN	HRSV A2	9878616
R429	F HRSV Long, A2	**19**, **20**, 56F, 57F, RSV19	VN	HRSV Long, A2	9878616, 1383404
K272	F BRSV strain 127	**B4** ^***^	VN	HRSV Long, A2	1383404
I432, K433, V447	HRSV B	**7.936**	VN	HRSV Long, A2	9658147
S436	HRSV B	**9.432**	VN	HRSV Long, A2	9658147
P389	HRSV Long	55F	VN	HRSV Long, A2	9658147

VN, virus neutralizing; Prot, *in vivo* protection; bold font indicates also cited by Swanson et al PNAS 2011

*human

**, rhesus

***bovine; Residues 262 and 272 also critical in seropositive humans [PMID:24499818]. *In vivo* protection for human mAbs represents those used in passive transfer experiments wherein the human-derived mAb is shown to protected recipients (most often rodents) from lethal challenge.

For the F protein, there are a total of 35 mAbs reported to date, representing 22 unique functional epitopes (unique site/Ab), the majority of which are discontinuous and often overlapping, clustering around specific regions (discussed further below). The IEDB defines an epitope as any unique molecular structure shown to interact with an antibody (see Methods). However, we acknowledge that overlapping binding sites can be considered as either separate epitopes or grouped together in some cases as part of a larger antigenic region, and this will be noted wherever possible going forward. For the G protein, far fewer discontinuous epitopes have been reported (7), and linear functional epitopes cluster very closely around the region containing residues ~140–200. All discontinuous G epitopes are also contained within this region.

### Structural analysis of the fusion protein epitopes

Previous work suggested that the majority of neutralizing epitopes may be elicited by the pre-fusion conformation of F [19, 26]; however, several neutralizing epitopes also recognize the post-fusion protein [19, 39–42]. Therefore, we investigated the overall repertoire of reported functional epitopes by mapping them onto the F protein’s pre-fusion and post-fusion three-dimensional (3D) structures. We were interested in evaluating the overall accessibility of the functional epitope sites and examining if these differed in pre- and post-F structures.

**Fig 2:**
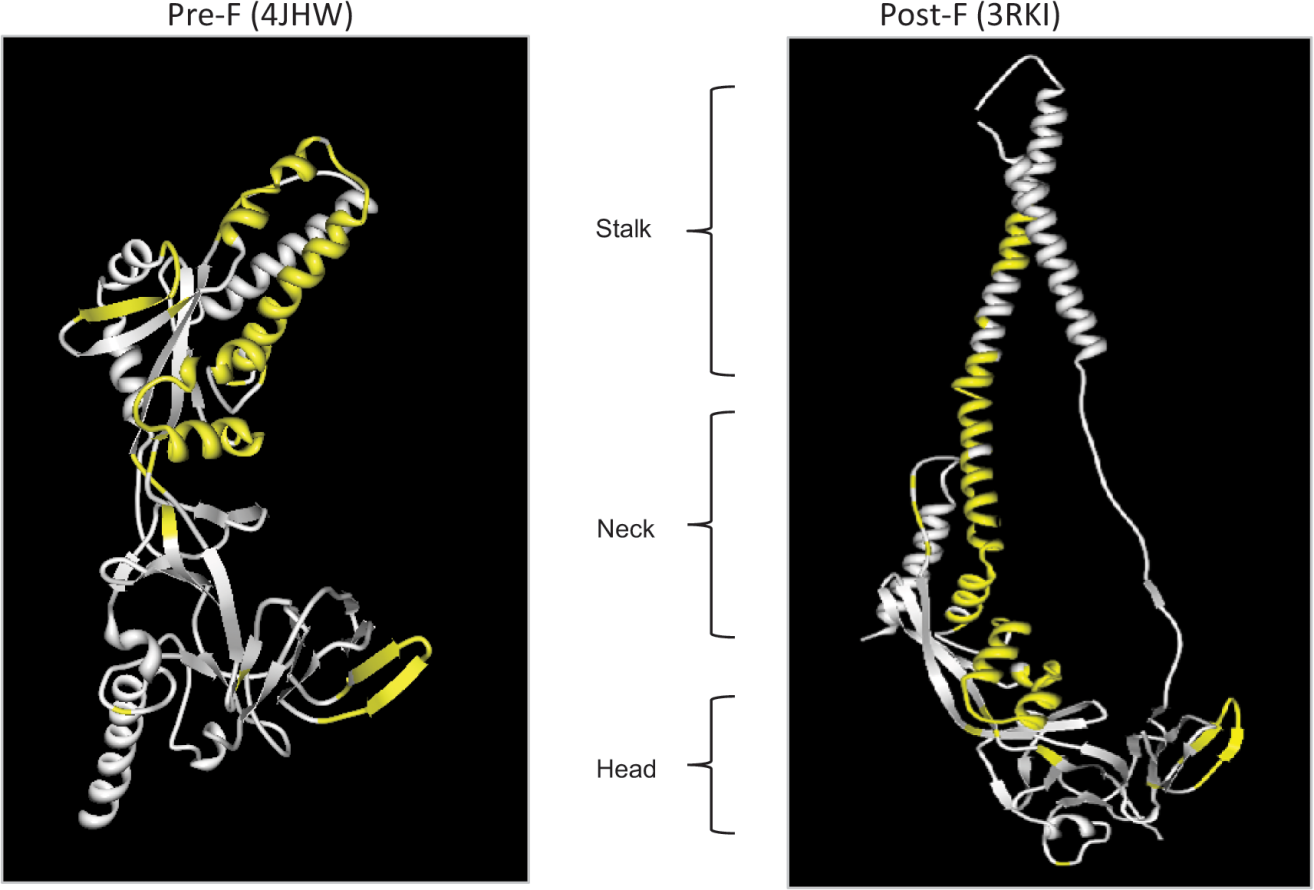
Functional B cell epitopes mapped to F protein. Functional epitopes, comprising 115 total residues, are mapped to the RSV F protein (epitopes are shown in yellow). Three dimensional structures 4JHW (pre-fusion) and 3RKI (post-fusion) were selected to visualize the location of all residues using the IEDB Homology Mapping Tool. This considers all functional (neutralizing and/or protective) linear and conformational epitopes described to date in the literature.

Using the IEDB’s Homology Mapping tool, epitope sites were mapped to the protein structures from the Protein Data Bank (PDB) of the pre-F [PDB: 4HJW] and post-F (Fig 2) [PDB: 3RKI] conformations. Functional epitopes are shown in yellow. Applying the structural blueprint provided by Colman and Lawrence defining the head, neck and stalk regions of F [43; defined on post-F structure], we were able to determine that of all 115 residues considered in this mapped functional antibody epitope subset, 21 were located in the head region (aa33-55 and 291–435), 59 in the neck region (aa56-105, 221–290 and 436–454) and 35 in the stalk (aa171-220). Here, the majority of epitope sites reported to date cluster to neck and stalk regions, with fewer sites located on the head or apex of the protein. In comparing the two states of the protein, it appears that in the post-F conformation in which the neck and stalk structure is formed, the solvent accessibility of some of these sites was decreased (Fig 2) [The lack of available PDB structures prevented a similar analysis for the G protein].

**Fig 3:**
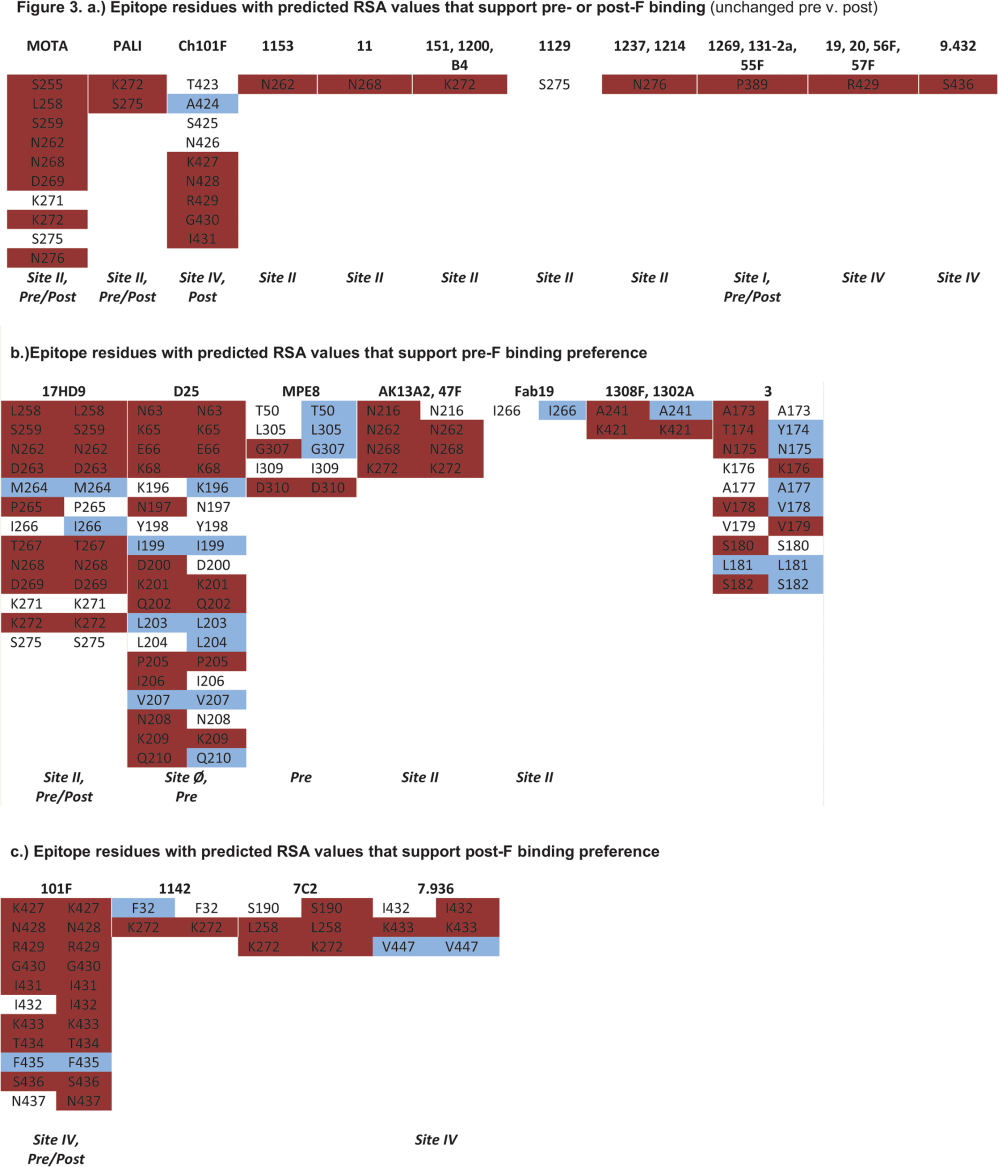
RSA scores for F protein (pre and post) for all mAbs. Calculated relative solvent accessibility (RSA) scores for each residue comprising the indicated mAb epitope for pre-F and post-F conformations are shown side-by-side for those that differed on pre- and post-F structures. Exposed residues (>40%) are shown in red, buried residues (0–7%) are shown in blue and half-exposed residues (7–40%) are un-colored. Included (if known) is indication of binding preference on pre-F, post-F or both and historical antigen binding site. Abbreviations: MOTA, motavizumab; PALI, palivizumab.

Thus to quantify and compare the change in accessibility from pre- to post-F, we calculated residues relative solvent accessibility (RSA) for each structure. Fig 3 shows RSA data for all monoclonal antibody epitope residues. In the figure, red denotes RSA of >40% (exposed), blue denotes 0–7% (buried) and no color 7–40% (intermediate-exposure), and for those residues with RSA values that differ on each conformation the results are displayed side-by-side for pre- versus post-F. Further, for each epitope we indicate where the mAb was reported to bind (either pre- or post-F if known), as well as the antigenic site (if known). Of the 22 unique functional epitopes considered, 11 showed no change in the residues RSA values from pre- to post-F (Fig 3A). These include, mAbs, mota, pali, Ch101F, 1153, 11, 151, 1200, B4, 1129, 1237, 1214, 1269, 131-2a, 55F, 19, 20, 56F, 57F, and 9.432. Another 11 show distinct patterns between pre- and post-F. Sites for mAbs 17HD9, D25, MPE8, AK13A2, 47F, Fab19, 1308F, 1302A and 3 show higher RSA values for pre-F (Fig 3B), while sites for mAbs 101F, 1142, 7C2 and 7.936 show higher RSA values for post-F (Fig 3C). These results may be of interest, for while the binding preference to pre-F has been established for mAbs D25 and MPE8, it is as yet undetermined for AK13A2, 47F, Fab19, 1308F, 1302A and 3. Similarly, the calculated RSA values suggest a binding preference for mAbs 1142, 7C2, and 7.936 to post-F. Thus we found correspondence in the RSA values for those mAbs previously shown to bind preferentially to a particular conformation and present RSA values suggestive of a binding preference for 14 functional mAbs that have yet to be differentiated. While it is not within the scope of the present analysis, the data presented here support future testing of this latter group of mAbs. Thus the binding preferences of the protective/neutralizing mAbs considered herein are not strictly determined for one conformation or the other. It is possible that these mAbs either bind pre-F or target epitopes accessible on both pre- and post-F conformations. Indeed, the RSA values of 15 of the 22 sites were either unchanged (no difference between pre and post) or were suggestive of a binding preference to F in its post-fusion conformation suggesting that epitopes to post-F are biologically relevant.

### Combined analysis of RSA values, sequence conservation and prediction outcomes for functional epitopes on F

The ability to map or identify B cell epitopes has been historically difficult, due in large part to the conformation-dependent nature of these determinants, which imposes numerous technical challenges related to the analysis of proteins in their native, 3-dimentional state. Also likely at issue, is the more practical problem of limited access to samples from human donors. Consequently, there has been increased interested in supplementing the current experimental methods with other analyses and bioinformatics tools. The idea being that these additional analytic tools may help decrease time and effort by identifying specific regions or sites of the greatest potential for activity that could then be tested empirically in the lab.

Using the tools at our disposal, we next sought to explore the relationship (if any) between solvent exposure (RSA values), evolutionary conservation scores and epitope prediction outcomes to determine if these measurements, when used in combination, could be discriminating factors in identifying true epitopes. Because the IEDB captures both positive and negative data, residues shown to be uniquely negative (non-binding) can be used to analyze truly non-immunogenic residues or regions. Here, RSA values and evolutionary conservation scores were calculated for non-binding, negative residues and compared to those calculated for all positive epitopes considered thus far from the subset of 22 mAbs (from Fig 3). This was done separately for each PDB structure available for pre-F (4JHW and 4MMS) and post-F (3RKI and 3RRR).

We found that on the pre-F PDB structures, residues from all positive residues (known epitopes) had a tendency to be solvent exposed in comparison to those from non-binding, negative residues, given the average RSA values of 43% and 12%, respectively (similar values were obtained for the PDB pre-F structures 4JHW and 4MMS). By contrast, on post-F PDB structures there was no difference observed in RSA values between positive residues (epitopes) and negative residues, with the average values for positive epitopes of 29% and 27% (again, similar result were observed for post-F PDBs 3RKI and 3RRR). Thus while positive and negative residues are discriminated on the pre-F structure by calculated RSA values, on post-F, both positive and negative residues are equally solvent accessible, showing RSA values indicating intermediate or partial exposure.

Next, we calculated residue conservancy scores for each PDB F structure and again made comparisons between known epitopes (positives) and negative residues. Here we found no significant difference between residue conservancy scores for positive and negative residues.

Lastly, we compared epitope prediction outcomes between the pre- and post-F conformations using two published B cell prediction methods, ElliPro [29] and Discotope (available on IEDB) [30, 31]. The predicted epitope scores calculated by these methods correlated well among pre-F conformations (r^2 = 0.52 for [PDB: 4JHW]; r^2 = 0.49 for [PDB: 4MMS]), but do not for post-F conformations. When we compared prediction outcomes for each method against known positive and negative residues we found that while there was no statistical significance, there was a correspondence with the observed scores, such that epitope prediction scores for known positive residues were consistently higher than that of negative residues. For example, the average ElliPro scores for positive residues were 0.63, 0.65, and 0.51 for PDBs 4JHW (pre), 4MMS (pre), and 3RKI (post), respectively, whereas the average scores for negative residues were 0.37, 0.37 and 0.28, respectively. Since both of these prediction methods (algorithms) take into account residue solvent accessibility, as well as overall protein structure, it is likely that the more globular pre-F conformation may be considered more suitable for the these specific methods than the elongated post-F conformation.

Thus the results of the combined analysis reveal a correspondence of RSA values and epitope prediction scores with positive and negative residues, but this was the case only when considering the pre-F structures. There was no observed tendency for the residues in functional epitopes to be more or less conserved than in reported non-binding, negative residues. No significant correlation was found between epitope prediction scores, RSA values and sequence conservation considered together for any structure (data not shown). All results for each of the analyzed structures are summarized in the S1 Table, which provides for each residue the conservancy score, RSA values, ElliPro and Discotope prediction scores.

### T cell epitope reactivity

Both CD4^+^ and CD8^+^ T cell subsets have been shown to play a critical role in protection from disease, and while involvement for each subset in disease enhancement has been implicated in certain animal models, in humans a balanced CD4^+^ Th_1_:Th_2_ cytokine response seems to promote development of neutralizing antibodies, while CD8^+^ cytolytic activity effects viral clearance from the lungs [44, 45]. Here we cataloged all human epitopes associated with *in vitro* IFNγ production (CD4/CD8), degranulation (perforin/granzyme B release), cytolytic activity (CTL), and *in vivo* protection. Unlike the case described for B cell/Ab epitopes above, we were not as limited in repertoire and could therefore select the human-specific subset of T cell data.

More than 50 peptides, from F, G, M2, M, NP and NS1, have been reported in the context of IFNγ (CD4^+^ and CD8^+^) and/or cytotoxicity (CD8^+^ T cell) [Tables 3–5]. NP represents the greatest source of CD8^+^ epitopes associated with IFNγ production and cytotoxicity, whereas epitopes associated with CD4^+^ IFNγ were most often reported from F. These data highlight the potential utility of NP as a vaccine antigen, and also show that in addition to being a major target of protective antibody responses, F is also a target of CD4^+^ and CD8^+^ T cell responses. Interestingly and perhaps not surprisingly, many of the epitopes described for CD8^+^ T cells are derived from antigens active within the cytoplasm (e.g. RNA binding role) [Tables 3 and 4], whereas many of the CD4^+^ T cell epitopes are derived from extracellular antigens active at the cell surface [Table 5].

**Table 3:** Epitopes that induce cytotoxicity in human CD8+ T cells.

**PMID**	Epitope	Position	Role/Site	Protein	Virus	MHC Allele
11024156	RARRELPRF	106–114	**EC**	**F2**	HRSV A2	HLA-B57
12667209	RELPRFMNYT	109–118	**EC**	**F2**	HRSV	HLA-A1
11024156	IAVGLLLYCKA	542–552	**TM/CP**	**F1**	HRSV A2	HLA-Cw12
17931110	QLLSSSKYT	16–24	**RB/CP**	**NP**	HRSV Long	HLA-A*02:01
17931110	KMLKEMGEV	137–145	**RB/CP**	**NP**	HRSV Long	HLA-A*02:01
12805430	AYGAGQVMLRWGVL	250–263	**RB/CP**	**NP**	HRSV A2	HLA-B8
12805430	AGQVMLRWGVLAKS	253–266	**RB/CP**	**NP**	HRSV A2	HLA-A2
12805430	QVMLRWGVL	255–263	**RB/CP**	**NP**	HRSV A2	HLA-B8
17931110	ILNNPKASL	303–311	**RB/CP**	**NP**	HRSV Long	HLA-A*02:01
10906229	NPKASLLSL	306–314	**RB/CP**	**NP**	HRSV	HLA-B7

CP: cytoplasm; TM: transmembrane; EC: extracellular; RB: RNA binding

**Table 4:** Epitopes that induce IFNγ production from human CD8+ T cells.

**PMID**	Epitope	Position	Protein	Role/Site	Virus	MHC Allele
15269378	AELDRTEEY	64–72	**M**	**VA/CP**	HRSV	HLA-B44
15269378	RLPADVLKK	151–159	**M**	**VA/CP**	HRSV	HLA-A3
15269378	IPYSGLLLV	195–203	**M**	**VA/CP**	HRSV	HLA-B51
15269378	YLEKESIYY	229–237	**M**	**VA/CP**	HRSV	HLA-A1
15755607	FVPCSICSNNPTCWAICKRIP	170–190	**G**	**HBD**	HRSV A2	HLA-Class I
15269378	LAKAVIHTI	41–49	**NS1**	**Inh IFN/CP**	HRSV	HLA-B51
11024156	RARRELPRF^#^	106–114	**F2**	**EC**	HRSV A2	HLA-B57
12667209	RELPRFMNYT	109–118	**F2**	**EC**	HRSV	HLA-A1
11024156	IAVGLLLYCKA^*#^	542–552	**F1**	**TM/CP**	HRSV A2	HLA-Cw12
17931110	QLLSSSKYT	16–24	**NP**	**RB/CP**	HRSV Long	HLA-A*02:01
12805430	KLCGMLLITEDANH	46–59	**NP**	**RB/CP**	HRSV A2	HLA-Class I
17931110	KMLKEMGEV	137–145	**NP**	**RB/CP**	HRSV Long	HLA-A*02:01
12805430	STRGGSRVEGIFAG	232–245	**NP**	**RB/CP**	HRSV A2	HLA-Class I
12805430	AYGAGQVMLRWGVL	250–263	**NP**	**RB/CP**	HRSV A2	HLA-Class I
12805430	AGQVMLRWGVLAKS	253–266	**NP**	**RB/CP**	HRSV A2	HLA-Class I
12805430	VMLRWGVLAKSVKN	256–269	**NP**	**RB/CP**	HRSV A2	HLA-Class I
12805430	AGFYHILNNPKASL	298–311	**NP**	**RB/CP**	HRSV A2	HLA-Class I
17931110	ILNNPKASL	303–311	**NP**	**RB/CP**	HRSV Long	HLA-A*02:01
12667209	NPKASLLSL	306–314	**NP**	**RB/CP**	HRSV	HLA-B7

Role/Site abbreviations are included to indicate the role of the protein antigen in viral pathogenesis. VA: viral assembly; CP: cytoplasm; TM: transmembrane; EC: extracellular; HBD: heparin binding domain 184–198; RB: RNA binding; Inh IFN; inhibits IFN-mediated antiviral response; SS: signal sequence/N-terminus.

**Table 5:** Epitopes that induce IFNg production in human CD4+ T cells.

**PMID**	Epitope	Position	Protein	Role/Site	MHC Allele
12502814	KANAITTILTAVTFCFAS	7–24	**F**	**SS**	HLA-DRB1*01:01; DRB1*04:01
12502814	TILTAVTFCFASGQNITE	13–30	**F**	**SS**	HLA-DRB1*01:01
12502814	GQNITEEFYQSTCSAVSK	25–42	**F**	**EC**	HLA-DRB1*04:01; DRB1*07:01
12502814	EFYQSTCSAVSKGYLSAL	31–48	**F**	**EC**	HLA-DRB1*07:01; DRB1*04:01
12502814	GYLSALRTGWYTSVITIE	43–60	**F**	**EC**	HLA-DQ5; HLA-DRB1*16:01
12502814	RTGWYTSVITIELSNIKE	49–66	**F**	**EC**	HLA-DRB1*04:07
12502814	SVITIELSNIKENKCNGT	55–72	**F**	**EC**	HLA-DRB1*07:01
12502814	DAKVKLIKQELDKYKNAV	73–90	**F**	**EC**	Class II-allele undetermined
12502814	IKQELDKYKNAVTELQLL	79–96	**F**	**EC**	HLA-DRB1*04:01
12502814	KYKNAVTELQLLMQSTPP	85–102	**F**	**EC**	HLA-DRB1*04:01
12502814	RELPRFMNYTLNNAKKTN	109–126	**F**	**EC**	Class II-allele undetermined
12502814	MNYTLNNAKKTNVTLSKK	115–132	**F**	**EC**	Class II-allele undetermined
12502814	NKAVVSLSNGVSVLTSKV	175–192	**F**	**EC**	HLA-DRB1*07:01
12502814	LDLKNYIDKQLLPIVNKQ	193–210	**F**	**EC**	HLA-DRB1*11:01
12502814	RLLEITREFSVNAGVTTP	229–246	**F**	**EC**	HLA-DRB1*11:01
12502814	REFSVNAGVTTPVSTYML	235–252	**F**	**EC**	HLA-DRB1*01:01
12502814	PITNDQKKLMSNNVQIVR	265–282	**F**	**EC**	HLA-DR3; DQ; DQ2
12502814	KKLMSNNVQIVRQQSYSI	271–288	**F**	**EC**	HLA-DQ; DR3; DQ2
12502814	EVLAYVVQLPLYGVIDTP	295–312	**F**	**EC**	HLA-DQ5; HLA-DQ6
12502814	VQLPLYGVIDTPCWKLHT	301–318	**F**	**EC**	HLA-DR; DQ
12502814	TDRGWYCDNAGSVSFFPQ	337–354	**F**	**EC**	HLA-DRB1*11:01;DRB1*13:01;DR3; DQ6
12502814	YDCKIMTSKTDVSSSVIT	391–408	**F**	**EC**	HLA-DRB1*04:01
12502814	SLGAIVSCYGKTKCTASN	409–426	**F**	**EC**	HLA-DRB1*15:01;DRB5*01; DRB1*07:01
12502814	KNRGIIKTFSNGCDYVSN	427–444	**F**	**EC**	HLA-DRB1*04:01
12502814	YYVNKQEGKSLYVKGEPI	457–474	**F**	**EC**	HLA-DRB1*01:01
12502814	VKGEPIINFYDPLVFPSD	469–486	**F**	**EC**	HLA-DRB1*15:01; DRB5*01
12502814	NAGKSTTNIMITTIIIVI	517–534	**F**	**EC/TM**	HLA-DQB1*0502; DPB1*16:01
12502814	LIAVGLLLYCKARSTPVT	541–558	**F**	**TM/CP**	HLA-DRB1*07:01
14747542	FHFEVFNFV	163–171	**G**	**HBD**	HLA-DPB1*04:02; DPB1*04:01
14747542	HFEVFNFVPC	164–173	**G**	**HBD**	HLA-DPB1*04:01;DPB1*04:02;DPB1*02:01; DPB1*02012
15755607	FVPCSICSNNPTCWAICKRIP	170–190	**G**	**HBD**	Class II-allele undetermined
14747542	RFAIKPME	248–255	**M**	**VA/CP**	HLA-DPB1*16:01

Role/Site abbreviations are included to indicate the role of the protein antigen in viral pathogenesis. VA: viral assembly; CP: cytoplasm; TM: transmembrane; EC: extracellular; HBD: heparin binding domain 184–198; RB: RNA binding; Inh IFN; inhibits IFN-mediated antiviral response; SS: signal sequence/N-terminus.

As a further analysis, a broad query was performed to identify all overlapping T and B cell epitopes that were recognized by children/infants and adults following (post) or during (acute) natural RSV infection (Table 6). These are indicated in Table 6 by asterisks. The table also includes the antigen name, epitope position, response frequency (number of respondents/number tested), the calculated RFscore (see Methods) and the function/location of the derivative antigen (if known) in viral pathogenesis. Here, ‘overlapping epitopes’ represent those residues that are recognized by both antibodies and T cells. In several instances we found that the exact same peptide could induce antibody and T cell response.

**Table 6:** ImmunomeBrowser analysis of B and T cell epitopes.

Epitope ID	Sequence	Cell	Ag/Position	R/T	RFscore	Function
64978	TLNKD**QLLSSSKYT**IQRSTG**	B	NP(11–30)	2/2	0.29 (0.71)	N-arm/CP
51436	**QLLSSSKYT**	T	NP(16–24)	2/10	0.06 (0.14)	N-arm/CP
61410	S**STRGGSRVEGIFAG**LFMNA**	B	NP(231–250)	2/2	0.29 (0.71)	NTD/CP
61861	STRGGSRVEGIFAG***	T	NP(232–245)	7/37	0.12 (0.07)	NTD/CP
48008	**PIVNKQSCSISNIETVIEFQQ**	B	F(205–225)	3/3	0.42 (0.58)	EC;HRA
48008	**PIVNKQSCSISNIETVIEFQQ**	T	F(205–225)	2/2	0.29 (0.71)	EC;HRA
59844	S**NIETVIEFQQ**KNNRLLEITREFSVNAGVTTPVSTYMLTNSELLSLINDMPITNDQKKLMS**	B	F(215–275)	5/9	0.31 (0.25)	EC;DI
44221	**NIETVIEFQQKNNRLLE**	B	F(216–232)	2/2	0.29 (0.71)	EC;DI
25806	**IEFQQKNNRLLE**ITRE	B	F(221–236)	2/2	0.29 (0.71)	EC;DI
53499	REFSVNAGVTTPVSTYMLTNSELLSLINDMPITNDQKKLMS**	B	F(235–275)	5/9	0.31 (0.25)	EC
139186	N**SELLSLINDMPITNDQKKLMSNN**	B	F(254–277)	4/4	0.50 (0.50)	EC
57525	**SELLSLINDMPITNDQKKLMSNNV**	B	F(255–278)	4/4	0.50 (0.50)	EC
57524	SELLSLINDMPITNDQKKLMS**	B	F(255–275)	4/10	0.20 (0.20)	EC
57525	**SELLSLINDMPITNDQKKLMSNNV**	T	F(255–278)	2/2	0.29 (0.71)	EC
17402	FPSDEF**	B	F(483–488)	2/2	0.29 (0.71)	EC;HRB
46727	NYYDPLV**FPSDEF**DAS*	T	F(476–491)	2/2	0.29 (0.71)	EC;HRB
125242	R**QNKPPSKPNNDFHFE**VFNFVP***	B	G(151–172)	22/51	0.34 (0.09)	Cys-noose
51730	**QNKPPSKPNNDFHFE**	T	G(152–166)	2/2	0.29 (0.71)	Cys-noose
18255	**FVPCSICSNNPTCWAICKRIP**	B	G(170–190)	6/6	0.59 (0.41)	Cys-noose
18255	**FVPCSICSNNPTCWAICKRIP**	T	G(170–190)	4/5	0.40 (0.40)	Cys-noose
94738	**WAICKRIPNKKP**	B	G(183–194)	2/2	0.29 (0.71)	HBD
100637	**WAICKRIPNKKP**G	T	G(183–195)	4/4	0.50 (0.50)	HBD
94429	**AICKRIPNKKPG**	B	G(184–195)	2/2	0.29 (0.71)	HBD
1862	**AICKRIPNKKPG**KKT	T	G(184–198)	2/2	0.29 (0.71)	HBD

R/T, number responded over number tested; RFscore, response frequency score; N-arm, N-terminal arm; NTD, N-terminal domain; CP, cytoplasmic, EC, extracellular; HBD, heparin binding domain; HRA/HRB, heptad repeats; DI, docking inhibition; Cys-noose, cysteine noose; bovine host*, natural infection in children/infants**, natural infection in adults***; bold indicates overlap.

Finally, we were interested in reported tetramer data, which are useful tools for studying T cell responses in the research and vaccine evaluation settings. A total of 11 RSV-derived tetramers were described, including epitopes restricted by both HLA class I and class II alleles (Table 7). All but one of these structures were also associated with a functional response (CTL, cytokine production, *in vivo* survival), making these potential reagents for use in evaluating candidate vaccine formulations for specific T cell response types.

**Table 7:** List of validated tetramers (epitope/allele combinations).

Tetramer	Antigen	Allele	Functional Response	PMID
HNYFEWPPHALLVRQ	M2 (25–39)	H-2-IAb	IFNγ	19264776, 24510524
NYFEWPPHALLVRQ	M2 (26–39)	H-2-IAb	NR	20686045
SYIGSINNI*	M2 (82–90)	H-2-Kd	CTL, Prot, IFNγ	19 references
YIGSINNI	M2 (83–90)	H-2-Kd	IFNγ	18816384
NAITNAKII*	M (187–195)	H-2-Db	CTL, IFNγ	17275872, 19153229, 20833834, 20686045, 21118816, 22144888
VYNTVISYI*	M2 (127–135)	H-2-Kd	CTL, IFNγ	17182672, 18662734
NKGAFKYIKPQSQFI	M (209–223)	H-2-IAb	IFNγ, IL-2, Prot	19264776, 20686045, 24510524
YLEKESIYY*	M (229–237)	HLA-A1	IFNγ	15269378, 15838799, 16301748
NPKASLLSL*	NP (306–314)	HLA-B7	CTL, IFNγ	15269378, 15838799, 10906229
KYKNAVTEL*	F (85–93)	H-2-Kd	CTL, Prot, IFNγ	11591747, 23015695, 24572813
CYLTDRARI	P (261–269)	H-2-Kd	IFNγ, Prot	16272314, 22940382

*Naturally eluted peptide; Prot, *in vivo* protection or survival; IFNγ, ELISPOT or ELISA; CTL, cytotoxicity assay; NR, None reported to date.

## Discussion

Herein we have provided an analysis of RSV specific immune epitope data with the goal of examining this body of work for its application to vaccine design and development. To this end, we first provided an overview of all available data, showing overall coverage and identifying critical knowledge gaps. Then we further analyzed the cumulative RSV epitope data from the standpoint of functional responses, and used a combinational approach to characterize antigenic structure and epitope location. This work represents a comprehensive analysis of RSV epitope data that provides an update for previous work by Anderson et al. [46].

Our initial assessment of the cumulative data revealed that the vast majority of epitope mapping has focused on just two antigens, F and G. While this is not surprising, it may be useful to pursue the expanded epitope mapping of this virus, not only to complete our understanding of these two critical proteins, but perhaps to also include additional antigens to more fully understand the immune response to all pertinent antigens having key roles in viral pathogenesis. Further, the balance of T cell versus antibody epitopes should be addressed, especially in light of increasing evidence from the literature regarding known correlates of protection, which suggests a role for both humoral and cellular responses in disease resolution. If it is found that combined antibody and T cell responses prove to be efficacious and safe, antigens such as NP might provide a potential target for inducing CMI, allowing the vaccine to generate the needed balance of both humoral and cellular responses. Indeed, our review of the T cell epitope data suggested there may be a need to incorporate multiple antigens (besides F) into candidate vaccines in order to optimize required response, but further empirical testing is warranted to make this determination.

Somewhat unexpected was the low number of human data; only 32% of the epitopes described to date are derived from human hosts, and of these a mere 3 epitopes were defined in humans describing neutralizing or protective activity. Thus our assessment of the totality of RSV epitope data is 1) that additional mapping of all proteins is warranted, including F and G, 2) a greater breadth in T cell, as well as B cell/antibody epitope repertories is needed, especially in humans and specifically using functional assays (neutralization, cytolysis, *in vivo* protection, etc.) and 3) future epitope discovery or mapping in the context of human disease, especially infants and young children would be beneficial to increase the characterization of immunity at the molecular level. Historically speaking, epitopes have not played a prominent role in RSV vaccine development; however, going forward, this information may help to more fully elucidate certain as yet poorly understood or partially characterized aspects of RSV immunobiology. While animal models of RSV have historically provided essential platforms through which we have gained valuable insights with respect to vaccine-enhanced disease and virus-induce airway hypersensitivity, and in which to test proof-of-principle for prophylactic and therapeutic agents [47], none truly recapitulate natural disease and therefore a better understanding the of human epitope repertoire will likely help hasten vaccine development.

As previously mentioned, the second part of our analysis builds on previous insights from McLellan et al. [18–20], and others, with regard to relationship of antigen conformation to epitope accessibility, and in particular in this report, epitopes known to be associated with protective responses. Extensive modeling by McLellan et al of the F protein using monoclonal antibodies revealed two important insights: 1) that there is structural preservation of important neutralizing epitopes in the post-fusion state, suggesting that this conformation would elicit neutralizing antibody responses and was therefore a useful target as vaccine antigen, and 2) that critical sites on pre-F are also protective. The work of McLellan et al. clearly attributes the greatest contribution to neutralizing response to the pre-fusion state of the F protein. Our review and analysis of the cumulative data (all reported to date) for ‘functional’ (neutralizing/protective) epitopes against F suggests that a majority of identified sites available for binding in the pre-F conformation are also accessible in the post-F conformation.

The current epitope data are as yet incomplete to determine the extent of neutralizing sites exclusive to either state because a comprehensive mapping of all potential neutralizing sites has not been performed. Thus, it is possible that in the course of natural infection sites do exist on both pre- and post-F and do contribute to disease resolution. Indeed, it possible that the epitope residues located within the ‘head and stalk’ regions of F function to prevent neutralizing on the pre-fusion conformation, whereas those epitopes found on the ‘apex or head’ function directly to inhibit contact with the host cell. However, no final conclusion can be drawn until further work comparing responses to both pre- and post-F states in humans is undertaken. To date, work by Sakurai et al. [48] looking at immune responses of people following natural infection describes a ‘envelop glycoprotein response dichotomy’ whereby antibody specificities to both immature (cell lysate) and mature (virus surface) forms of F exist, and should therefore be considered for the purpose of vaccine development.

Going forward, it may be important to establish to what extent epitopes on each state contribute to RSV disease resolution in humans, especially the target population, children 6 months to 2 years of age, as these data are yet unavailable in the current literature. In fact as noted above, very little epitope analysis of humans during and following infection disease has been published, especially neutralizing sites specific to this host (infants and adults). This is surprising given the ubiquitous nature of this pathogen, by comparison to influenza A virus as an example. To date, there are 379 assays defining more than 50 flu A neutralizing sites in humans captured from the peer-reviewed literature in the IEDB. Historically, we do know that vaccine-induced partial immunity against RSV has been a double-edge sword. If we can more fully characterize the nature of protection at the molecular level against RSV we may, for example, be better positioned to answer the question, ‘are the majority of the neutralizing epitopes present on pre-F, or are these critical epitopes located on both pre- and post-F?’ The answer may influence the chosen vaccine modality (recombinant vector expressing whole antigen, plasmid DNA, live-attenuated, purified protein with adjuvant, etc.) and its complexity from a formulation standpoint.

As part of our investigation into the nature of protective epitopes, we sought to determine to what extent, if any, functional antibody and T cell epitopes residues overlapped. We reasoned that such analysis was relevant to vaccine design and development since evidence to date demonstrates that the generation of a combined humoral and cellular response is optimal against RSV. Thus considering the antibody data together with the T cell data may be of value for vaccine development, especially if the aim is to develop, for example, a subunit vaccine (say, 1–2 antigens) that targets both humoral and cellular immunity. Interestingly, we found that there was overlap between certain Ab and T cell sites (CTL/IFNγ epitopes with neutralizing linear and discontinuous epitopes). Moreover, some of these overlapping residues occur within important structural/functional features of the respective antigens. This was shown to be true for linear (polyclonal and mAbs) and discontinuous antibody epitopes (mAbs), as well as both CD4^+^ and CD8^+^ epitopes. Furthermore, many of these same overlapping epitopes residues are also recognized in the context of natural infection. However, we acknowledge that antibody and T cell responses represent functionally distinct processes and concede that the data are as yet insufficient to elucidate a true relationship. It may simply be that functionally important antigens are made in abundance and are therefore likely “seen” more frequently by B and T cells. We hope that ultimately it will be feasible to apply this layering approach using subsets of data selected for high stringency (assays/correlates, etc.) to define epitopes/antigens that are “heavy hitters” from an immunological perspective.

Finally, we also gained some insights into B cell epitope prediction, which suggested that future B cell/antibody prediction tools may incorporate the identification of solvent accessibility scores, as well as of a protein’s features of structural significance. Indeed, there are now databases devoted to protein structure that may be useful in this regard (e.g. ProFunc, PDB, UniProt) [49]. Our data support the premise that epitope location (known positives; not merely predicted) is not solely defined by exposure, as we observe that not all exposed residues are epitopes, and that conversely less exposed and moderately buried residues are often part of functional epitopes. Perhaps epitope prediction in the future will benefit from the inclusion of other factors, such as post-translational modification (e.g., glycosylation). Further, we find that functional epitopes tend to cluster, not unexpectedly around sites known to be involved in antigen function and/or activity. Ultimately, it is our hope that this work will provide the basis for further RSV-specific epitope discovery, as well as future investigation into the nature of antigen conformation in immunogenicity in humans.

## Supporting Information

S1 Table This table provides the results summary of all methods reported for all residues (epitopes) considered: conservancy scores, RSA values, as well as ElliPro and Discotope prediction scores. (XLSX)
